# Identification of RT-qPCR reference genes suitable for gene function studies in the pitaya canker disease pathogen *Neoscytalidium dimidiatum*

**DOI:** 10.1038/s41598-022-27041-w

**Published:** 2022-12-26

**Authors:** Meng Wang, Zhouwen Wang, Shuangshuang Wei, Jun Xie, Jiaquan Huang, Dongdong Li, Wenbin Hu, Hongli Li, Hua Tang

**Affiliations:** 1 Hainan Yazhou Bay Seed Laboratory, Sanya Nanfan Research Institute of Hainan University, Sanya, 572025 China; 2 College of Tropical Crops/Hainan Key Laboratory for Sustainable Utilization of Tropical Bioresources, Hainan University, Haikou, 570228 China; 3 College of Life Science, Hainan University, Haikou, China; 4 Tropical Crops Genetic Resources Institute, Chinese Academy of Tropical Agricultural Sciences, Haikou, 571700 China

**Keywords:** Microbiology, Molecular biology, Plant sciences

## Abstract

*Neoscytalidium dimidiatum* is the main causal agent of pitaya canker. Most studies of virulence and pathogenicity genes have measured expression levels using real-time quantitative polymerase chain reaction (RT-qPCR). Suitable reference genes are essential for ensuring that estimates of gene expression levels by RT-qPCR are accurate. However, no reference genes can be robustly applied across all contexts and species. No studies to date have evaluated the most effective reference genes for normalizing gene expression levels estimated by RT-qPCR in *N. dimidiatum*. In this study, RT-qPCR data for individual candidate reference genes were analyzed using four different methods: the delta Ct method and the geNorm, NormFinder, and BestKeeper algorithms. We evaluated the utility of eight candidate reference genes (*18S rRNA, Actin (1), Actin (2), Actin, GAPDH (1), GAPDH (2), UBQ*, and *Tubulin*) for normalizing expression levels estimated by RT-qPCR in *N. dimidiatum* at different developmental stages, at different temperatures, and during interaction with pitaya*.* All candidate reference genes were suitable for gene expression analysis except for *Actin (2)*. *Tubulin* and *Actin (1)* were the most stably expressed reference genes under different temperatures. *Actin (1)* and *Actin* were the most stably expressed reference genes in *N. dimidiatum* at different developmental stages. *Tubulin* and *UBQ* were the most stably expressed reference genes during interaction with pitaya. *Actin and 18s rRNA* were the most stably expressed across all experimental conditions. Subsequently, *Tubulin* and *UBQ* were further investigated in analyses of pectinase expression during the pitaya–*N. dimidiatum* interaction. Our results provide insights that will aid future RT-qPCR studies of gene expression in *N. dimidiatum*.

## Introduction

*Neoscytalidium dimidiatum* is the main causal agent of pitaya canker in southern China^1^. Pitaya canker has spread rapidly worldwide and has resulted in major economic losses^2^. Pitaya canker has become one of the most serious diseases affecting pitaya production. Previously, we have shown that pitaya canker cannot be completely eliminated by chemical treatment with traditional fungicides because the pathogen penetrates the epicuticular wax of the host; consequently, once a plantation is infected with *N. dimidiatum*, ulcers can reappear when climate conditions are suitable^3^. The mechanisms underlying pitaya–*N. dimidiatum* interactions and pathogen infection remain unknown.

The plant cell wall, which represents the first protective barrier against pathogens, is a composite material made of cellulose microfibrils embedded in a matrix of hemicellulose and pectin^4^. Cell wall-degrading enzymes secreted by the fungus might play an important role during the infection process. For example, lytic polysaccharide monooxygenases can recognize and degrade pectin, and silencing of the genes encoding these enzymes in *Phytophthora infestans* inhibits infection of potato^5^. Clarifying the role of pectinases in *N. dimidiatum* infection requires gene expression analysis.

Real-time quantitative PCR (RT-qPCR) is the most used method for detecting gene expression patterns^6–8^. The selection of appropriate reference genes is important for ensuring that estimates of gene expression are accurate^9^, as the use of inappropriate reference genes can result in inaccurate results^10^. Quantitative evaluations of gene expression in *N. dimidiatum* during infection can be used to identify the genes involved in virulence or pathogenesis. The use of one or more stable reference genes is the most common method for normalizing RT-qPCR data. In RT-qPCR analysis, normalization of the data requires internal control gene(s) that display uniform expression under various biological conditions^11^. Therefore, a suitable reference gene(s) needs to be selected based on specific sets of experimental conditions relevant to the study objective. Validations of internal reference gene stability have been performed in some fungi, including *Valsa mali* var. *mali*^12^, *Magnaporthe oryzae*^13^, *Xanthomonas oryzae* pv. *oryzae*^14^, and *Ustilaginoidea virens*^15^. However, no studies to date have systematically screened for stably expressed reference genes for RT-qPCR analysis in *N. dimidiatum*.

In this study, eight candidate reference genes (*18S rRNA, Actin (1), Actin (2), Actin, GAPDH (1), GAPDH (2), Tubulin*, and *UBQ*) were screened in *N. dimidiatum*. As *Actin (2)* exhibited non-specific amplification, we evaluated the utility of *18S rRNA, Actin (1), Actin, GAPDH (1), GAPDH (2), Tubulin*, and *UBQ* as reference genes for RT-qPCR in *N. dimidiatum* at different stages of development, under different temperatures, and during interaction with pitaya. Three Excel add-ins (i.e., BestKeeper, geNorm, and NormFinder) were used to evaluate the stability of the expression of these genes^16–19^. The suitable reference genes identified will facilitate future studies of gene function in *N. dimidiatum*.

## Results

### Selection of candidate reference genes

Eight candidate reference genes from the *N. dimidiatum* genome were selected based on non-redundant functional annotations. The length of the primers was approximately 20 bp, the size of the amplified products ranged from 106 bp for *GAPDH (1)* to 185 bp for *Actin* (Table 1).

**Table 1:** Primers of the candidate reference genes use in this study.

Gene abbrev.	Gene ID	Primer SEQUENCE (5′→3′) (Forward/Reverse)	TM (°C)	Size (bp)
*Actin (1)*	EVM0009841.1	CCCAAGTCCAACCGTGAGAA	60.5	165
		CGTAGATGGGGACCAAGTGAGT		
*Actin (2)*	EVM0008780.1	AGAACTTCCCCGACCACCA	59.7	185
		CATGTCATCCCATCGCTTCAC		
*18S rRNA*	EVM0010269.1	TCATCGCGGTGGAAATGG	60.5	156
		TCTGGTAGGGCGTGTTGGAG		
*GAPDH (1)*	EVM0006823.1	TACCTTCAGCGGGTCAGTCG	60.9	106
		CTTTGTCCCAGTTGATGTTTGC		
*GAPDH (2)*	EVM0001473.1	ATGTTCGTCATGGGCGTCA	59.9	174
		TCTGGGTGGCAGTGTAGGAGT		
*UBQ*	EVM0010754.1	GGGAAGCGTAGGGAAGAGGA	60.7	126
		GAGCAGGATCAAAGCAGAGTGA		
*Tubulin*	EVM0008969.1	GAACCTGAACCGCCTGATTG	60.2	136
		GACCAGAGGGAAGTGGATACG		
*Actin*	EVM0008636.1	ATCCCTGGAAGCGTCAACTG	59.0	133
		CAACCAGACTGAACCCGACC		
*pectinase*	ND3060	ACCGCTCTTGCGGTAATTG	58.7	112
		TCGCCGTAGTTGGAGTTGAT		

Gene ID, primer pair sequences and characteristics, and expected amplicon sizes. Gene abbreviations are used to refer to each candidate reference gene.

### Expression profiles of candidate reference genes

**Figure 1:**
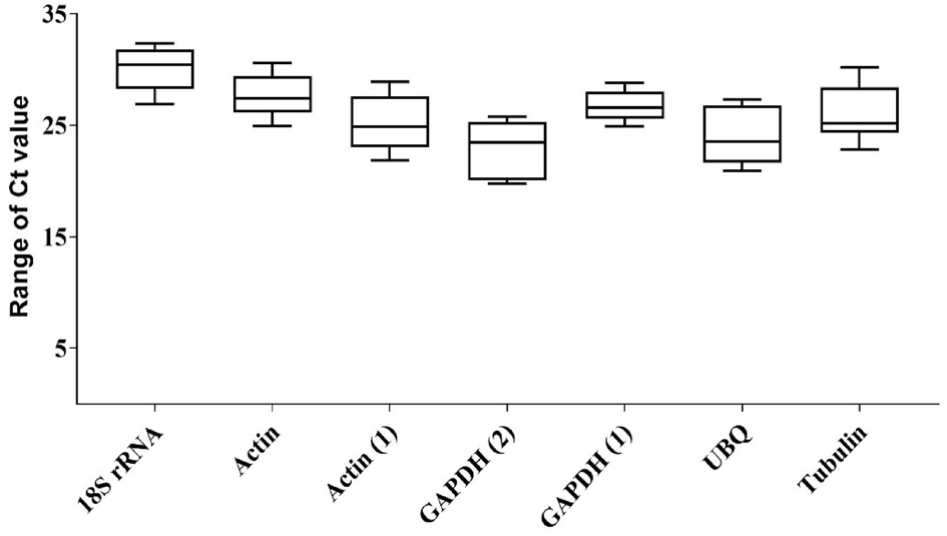
Ranges of expression of the seven selected reference genes in all analyzed samples. The whisker box plots represent the cycle threshold numbers (Ct value) of the seven reference genes. The horizontal lines inside the boxes are the median values, and the whiskers of the boxes indicate the minimum and maximum.

To test the specificity of the RT-qPCR primers, PCR was used to amplify the sequences of the eight candidate genes. All primers except *Actin (2)* amplified a single PCR product of the expected size according to 1.5% gel electrophoresis and melting curve analysis (Figs. S1, S2). Ct values for the seven candidate genes showing specific amplification (i.e., excluding *Actin (2)*) ranged from 19.80 to 31.62. The expression of *GAPDH* (2) was the highest, and the expression of *18S rRNA* was the lowest among all candidate genes. Variation in expression was the lowest for *GAPDH (2)* among all candidate genes (mean Ct value ± SD = 26.77 ± 1.39). Variation in expression was the highest for *18S rRNA* and *Actin (1)* among the seven candidate genes (23.97 ± 2.61 and 22.97 ± 2.67, respectively) (Fig. 1, Table 2).

**Table 2:** Variation in the expression stability of seven candidate reference genes determined using the 2^−ΔΔCt^ method, geNorm, Bestkeeper, and Normfinder.

Rank	Delta Ct			geNorm		Bestkeeper		Normfinder	
	Gene abbrev.	Average *C*t	SD	Gene abbrev.	M	Gene abbrev.	CV	Gene abbrev.	SV
1	*GAPDH (2)*	26.77	1.39	*Actin*	0.084	*GAPDH (1)*	4.00	*Actin*	0.014
2	*UBQ*	27.67	1.96	*18S rRNA*	0.094	*Actin*	5.19	*18S rRNA*	0.025
3	*Tubulin*	30.08	2.04	*Tubulin*	0.096	*18S rRNA*	5.35	*Tubulin*	0.040
4	*Actin*	25.2	2.56	*Actin (1)*	0.109	*Actin (1)*	7.60	*GAPDH (2)*	0.059
5	*GAPDH (1)*	26.02	2.61	*GAPDH (2)*	0.116	*Tubulin*	7.68	*Actin (1)*	0.059
6	*18S rRNA*	23.97	2.61	*UBQ*	0.129	*UBQ*	9.18	*UBQ*	0.069
7	*Actin (1)*	22.97	2.67	*GAPDH (1)*	0.181	*GAPDH (2)*	10.17	*GAPDH (1)*	0.117

RT-qPCR data were analyzed at different developmental stages, at different incubation temperatures, and during interaction with pitaya*.*

### Expression of candidate genes in *N. dimidiatum* at different temperatures

**Figure 2:**
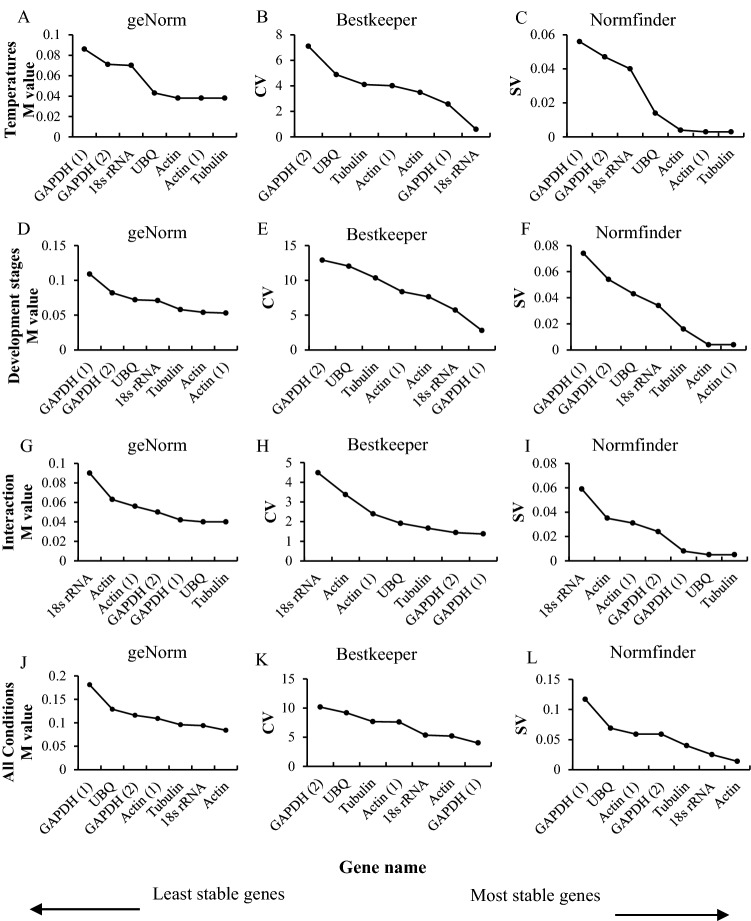
Rankings of reference gene expression stabilities determined by RT-qPCR followed by geNorm, Bestkeeper, and Normfinder algorithm. (**A**–**C**) correspond to different temperatures; (**D**–**F**) *N. dimidiatum* at different developmental stages*;* (**G**–**I**) correspond to pitaya*–N. dimidiatum* interaction; (**J**–**L**) correspond to all conditions (at different developmental stages, at different temperatures, and during the pitaya–*N. dimidiatum* interaction.

The expression levels of the seven candidate genes in *N. dimidiatum* at different temperatures and developmental stages were first determined by RT-qPCR, and their expression stabilities were assessed using three statistical algorithms (Fig. 2). The average expression stability (M-value) of all genes was calculated by geNorm. Genes with the lowest M-value are considered the most stable. In our analysis, all genes had M-values below the geNorm threshold of 1.5. The expression of *Tubulin* and *Actin (1)* were the most stable under different temperature treatments. Analysis of RT-qPCR data using the BestKeeper algorithm indicated that the expression of *18S rRNA* was the most stable at different temperatures. The NormFinder algorithms indicated that the expression of *Tubulin* and *Actin (1)* was the most stable at different temperatures (Fig. 2A–C).

### Expression of the candidate genes at different developmental stages

To determine the expression of the seven candidate genes at different developmental stages, we analyzed their expression at 3 days, 5 days, and 10 days. Analyses of the RT-qPCR data using the geNorm algorithm revealed that the expression of *Actin (1)* and *Acin* were the most stable at different developmental stages. Analysis of RT-qPCR data using the BestKeeper algorithm revealed that the expression of *GAPDH (1)* was the most stable at different developmental stages. Based on the NormFinder algorithm, *Actin (1)* and *Acin* were the most stably expressed gene at different developmental stages (Fig. 2D–F).

### Expression of selected genes during interaction with pitaya

Analysis of RT-qPCR data using geNorm or NormFinder revealed that the expression of *Tubulin* and *UBQ* were most stable during interaction with pitaya. However, the expression of *GAPDH (1)* was determined to be the most stable when the BestKeeper algorithm was used to analyze the RT-qPCR data (Fig. 2G–I).

### Suitability of the candidate genes

In addition to identifying the optimal reference genes for each treatment, we used three different algorithms to estimate the overall suitability of these reference genes to be used across all experimental contexts (i.e., at different temperatures, different developmental stages, and during interaction with pitaya). Analysis with the geNorm and NormFinder algorithm revealed that the optimal reference gene was *Actin*, and analysis with the BestKeeper algorithm revealed that *GAPDH (1)* was the most suitable reference gene (Fig. 2J–L).

### Determination of the optimal number of reference genes needed for the normalization of RT-qPCR data

**Figure 3:**
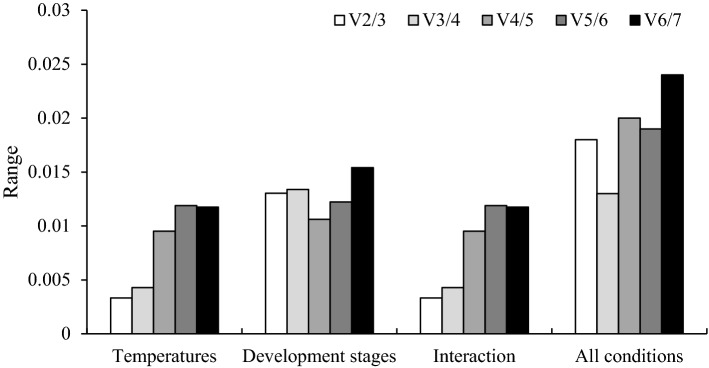
Pairwise variation (V) analyses of the seven candidate reference genes using the geNorm algorithm. “All conditions” corresponds to the comparison of *N. dimidiatum* at different developmental stages, at different growth temperatures, and during interaction with pitaya.

A single reference gene is often insufficient for accurately quantifying levels of gene expression. It is frequently necessary to use two or more than two reference genes to ensure the accurate normalization of gene expression levels^9^. To determine whether two or more *N. dimidiatum* reference genes are needed to normalize the RT-qPCR data, pairwise variations were determined using the geNorm algorithm. The optimal number of reference genes can be determined by using the pairwise variation value (Vn/Vn + 1, V-value) as the normalization factor for reference genes. Additional (n + 1) reference genes are necessary to normalize the genes if the V-value is higher than the threshold of 0.15. The ratio of V2/V3 was lower than 0.15, indicating that the optimal number of reference genes was two. The results in Fig. 3 showed that all V-values were less than 0.15, indicating that the addition of a third gene for correction was unnecessary. That is, two reference genes are needed as internal controls for normalizing gene expression data in *N. dimidiatum* at different temperatures (*Tubulin/Actin (1)*), at different developmental stages (*Actin/Actin (1)*), during interaction with pitaya (*Tubulin/UBQ*), and at all conditions (*18s rRNA/Actin*) (Fig. 3).

### Reference gene validation

**Figure 4:**
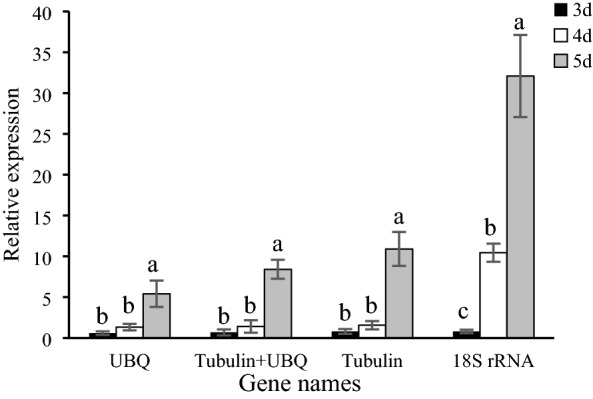
Relative expression of the pectinase gene in *N. dimidiatum*. *Tubulin/ UBQ*/ *Tubulin* + *UBQ* were used as one or two stable reference genes. The expression of the reference gene *18S rRNA* was the least stable.

Pectin forms an acidic polysaccharide network and makes up the scaffold of the middle lamella that binds plant cells to one another. Therefore, a large amount of pectinase is often secreted in the early stage of infection to dissolve the cell wall of plants. Once the plant cell wall is breached, the pathogen secretes intracellular effector proteins that manipulate the host and its defense mechanisms^20^. To determine the reliability of the two optimal reference genes identified (*UBQ* and *Tubulin*) and the least optimal reference gene (*18S rRNA*) during interaction with pitaya, we used RT-qPCR to detect the expression of the pectinase gene (*ND3060*) during the pitaya–*N. dimidiatum* interaction (Fig. 4, Table S1). The total RNA was extracted from pitaya infected with *N. dimidiatum* at 3 days, 4 days, and 5 days, and used to synthesize the first strand of cDNA, which will be used as the template of RT-PCR, and relative expression levels were calculated using the 2^−△△Ct^ method. When *Tubulin* and *UBQ* were used as internal controls, the expression of pectinase gradually increased on 3 days, 4 days, and 5 days. This finding was consistent with the pattern observed under *N. dimidiatum* infection of pitaya. When pitaya was infected by *N. dimidiatum* from 5 to 7 days, the expression of disease resistance-related genes of pitaya was highest^3^. However, *18S rRNA* overestimated the expression of pectinase on 4 d.

## Discussion

Pitaya canker is an emerging global disease that poses a significant threat to pitaya production^21^. Currently, little is known about the virulence functions of *N. dimidiatum*^3^. Most functional analyses of virulence or pathogenicity genes that involve gene expression analysis are performed using RT-qPCR^12,22,23^. A suitable internal reference gene is essential for correcting for errors in RNA quantity, reverse transcription efficiency, and the stability of different reaction channels of the PCR instrument and standard operating mode and determining the expression level of the target gene^15,24^. Housekeeping genes such as *Tubulin*, *Actin*, and *18S rRNA* are often used as reference genes. However, these reference genes show expression instability in various species, at different developmental stages, or in different tissues^8,9,14,18^. Therefore, the reference genes most appropriate for specific sample types and experimental conditions need to be selected. However, no studies to date have systematically evaluated the set of reference genes most suitable for use in *N. dimidiatum*.

In this study, we evaluated the stability of eight commonly used housekeeping genes for specific amplification analysis and found that *Actin (2)* exhibited non-specific amplification. Therefore, we used seven housekeeping genes for specific amplification analysis. A series of experiments were performed as an internal control for the analysis of differential gene expression in *N. dimidiatum*. We found that *GAPDH (2)* was the most abundant gene compared with the other six candidate genes tested based on Ct values. However, this gene was not stably expressed in different tissues of *N. dimidiatum*. In addition, its expression during interaction with pitaya was only moderately stable at different developmental stages of *N. dimidiatum* and under different temperatures. These findings suggest that *GAPDH (2)* is not the most suitable reference gene for RT-qPCR.

*Actin* is an important cytoskeletal protein in cells. It is highly conserved across different species, and it is also the most common reference gene used for RT-qPCR^25^. We comprehensively analyzed the interaction of the *Actin* gene with pitaya at different temperatures and different developmental stages. The expression of *Actin (1)* was the most stable at different developmental stages and temperatures, but its expression was not stable under other sets of experimental conditions. Thus, this gene could be used as a reference gene for *N. dimidiatum* under specific experimental conditions.

*Tubulin* is the basic building block of microtubules and plays a role in many fundamental cellular processes, such as the maintenance of cytoskeletal structure^26^. We found that the expression of *Tubulin* was stable during interaction with pitaya and under different temperatures; the expression stability of *Tubulin* was intermediate among all candidate genes analyzed. Therefore, this gene is most suitable as a reference gene for RT-qPCR during interaction with pitaya (Fig. 2).

*UBQ* (*Polyubiquitin*) is a ubiquitin gene^27^. geNorm was used to analyze the expression of genes during the pitaya–*N. dimidiatum* interaction at different periods; two reference genes are needed to ensure the accuracy of expression levels calculated during the pitaya–*N. dimidiatum* interaction. During the interaction period, the expression of *Tubulin* was the most stable, followed by *UBQ*. Therefore, the use of *Tubulin* and *UBQ* to normalize RT-qPCR taxa can maximize the accuracy of gene expression estimates.

Pectinase can destroy the cell wall of plants^20^. When *N. dimidiatum* infects pitaya, it secretes a large amount of pectinase to destroy the cell wall of pitaya in the early stage of infection, which allows it to obtain nutrients. Therefore, the expression of the pectinase gene *ND3060* was observed in the early stage of *N. dimidiatum* infection. When a single gene or combination of genes (*Tubulin* and *UBQ*) was used as an internal control, the expression of pectinase gradually increased on 3 days, 4 days, and 5 days of *N. dimidiatum* infection of pitaya, which was consistent with the expression patterns of pitaya during infection. These results indicate that *Tubulin* is the most stably expressed gene during the pitaya–*N. dimidiatum* interaction, and the use of both *Tubulin* and *UBQ* can increase the accuracy of estimates of the expression of genes related to pitaya–*N. dimidiatum* interaction.

## Conclusion

This study represents the first attempt to select a set of commonly used candidate reference genes in *N. dimidiatum* at different developmental stages, at different temperatures, and during interaction with pitaya for the normalization of gene expression data using RT-qPCR*. Tubulin* and *Actin (1)* were the most stably expressed reference genes under different temperatures. *Actin (1)* and *Actin* were the most stably expressed reference genes in *N. dimidiatum* at different developmental stages. *Tubulin* and *UBQ* were the most stably expressed reference genes during interaction with pitaya. *18s rRNA* and *Actin* were the most stably expressed across all experimental conditions.

## Materials and methods

### Plant material, growth conditions, and infection

**Figure 5:**
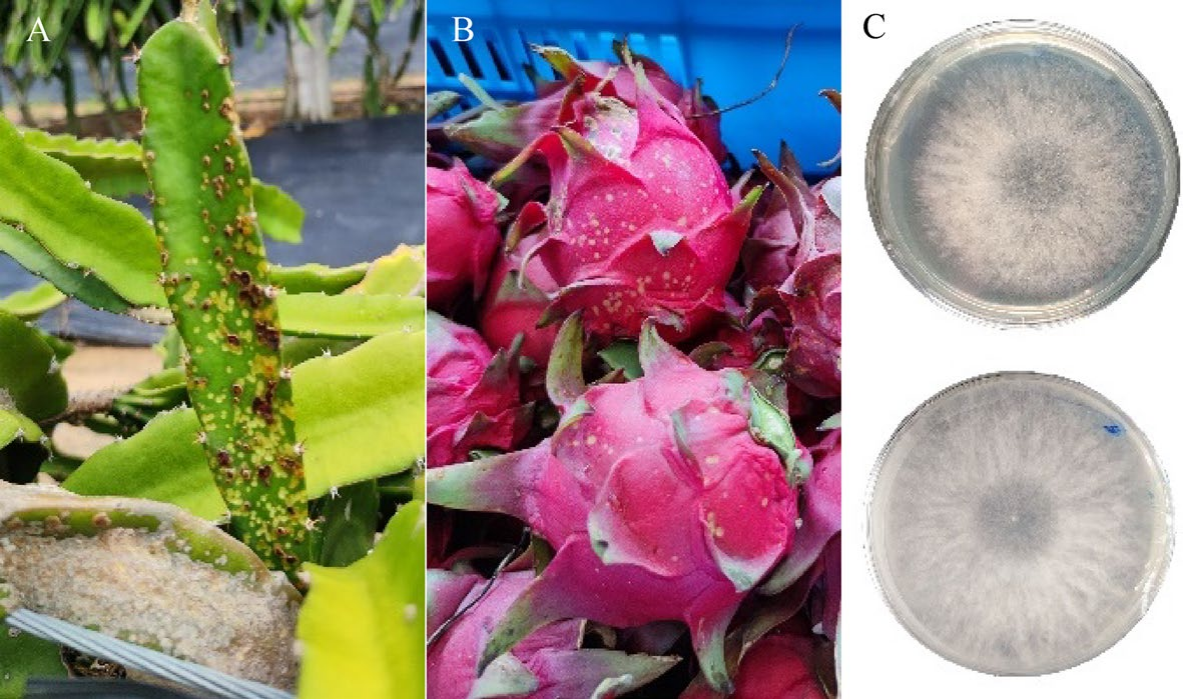
(**A**) Diseased stems in a field plantation; (**B**) diseased pitaya fruits; (**C**) morphological characteristics of *N. dimidiatum.*

The pitaya was sampled from the pitaya germplasm nursery of Hainan University located at Banqiao Town, Dongfang City, Hainan Province, China (longitude: 108°68′ E, latitude: 18°79′ N). The pitaya germplasm nursery can continue to provide us with experimental materials for further experiments. The voucher specimen of pitaya in our study was collected and identified by Professor Hua Tang, it has been deposited in pitaya germplasm nursery as number ‘HD-A38-10’ and with herbarium name ‘Jindu NO. 1. It is a publicly available herbarium by some procedure. The ‘Jindu NO. 1’ is an important and major pitaya variety in pitaya production, it was bred and named by Mr. Jindu Wang. The use of plants in the present study complies with international, national and/or institutional guidelines. *N. dimidiatum* was isolates from diseased pitaya stem tissues and stored at -80 °C at Hainan Key Laboratory for Sustainable Utilization of Tropical Bioresources, Hainan University, Haikou, China^2^ (Fig. 5). The hyphae were collected from *N. dimidiatum* by culture on PDA medium at 15 °C, 28 °C, and 37 °C for 5 days; *N. dimidiatum* was cultured on PDA medium at 28 °C, and the hyphae were collected at 3 days, 5 days, and 10 days.

Pitaya twigs with the same growth state were collected from the pitaya field; their surfaces were sterilized with 75% ethanol in the laboratory of the School of Tropical Crops, Hainan University, rinsed with tap water, and then dried. *N. dimidiatum* that had been cultured in PDA liquid medium for a week was collected, filtered through three lens cleaning tissue, and diluted with sterile water to a concentration of 1 × 10^5^ spores. The spore suspension was evenly sprayed on the surface of pitaya, and the twigs were kept moist at room temperature. Notably, all the above treatments had three biological replicates. The remaining pitaya twigs were sprayed with sterile water as a control. Lastly, collect the obviously diseased parts of pitaya infected by *N. dimidiatum* at the 3 days, 4 days and 5 days, frozen in liquid nitrogen, and stored at − 80 °C.

### RNA extraction and cDNA synthesis

Total RNA from pitaya–*N. dimidiatum* interaction samples was extracted following the protocol of Xu et al.^2^. Harvested mycelia were ground to a fine powder in liquid nitrogen using a pre-cooled mortar and pestle. Total RNA of *N. dimidiatum* from the samples was extracted using TRI Reagent RNA Isolation Reagent (Sigma, Shanghai, CHN) per the manufacturer’s instructions and then treated with RNase-free DNase I at 37 °C for 30 min. Purification with TRI Reagent RNA Isolation Reagent was repeated to remove DNase I. RNA quality was analyzed by 2.0% agarose gel electrophoresis, and RNA integrity was assessed using a NanoPhotometer (Implen, GER). Only RNA samples with an A260/A280 ratio of 1.8–2.2 and an A260/A230 ratio > 1.8 were used for further analysis. cDNA was synthesized from 1.0 μg of total RNA samples using a HiScript III cDNA Synthesis Kit per the manufacturer’s instructions (Vazyme Biotech Co., Ltd., Nanjing, China, Cat. No. R333).

### Candidate gene selection and primer design

The expression of eight candidate reference genes (*18S rRNA, Actin (1), Actin (2), Actin, Tubulin, GAPDH (1), GAPDH (2),* and *UBQ*) was analyzed. The specific primers (Table 1) were designed using Primer Premier 5.0 with the following parameters: melting temperature (Tm), 48–61 °C; GC percent, 40–60%; primer lengths, 18–22 bp; and product lengths, 100–200 bp.

To confirm that the correct region was amplified by the selected primers, primer pairs were used to prime amplification in 100 ng of the first-strand cDNA in an end-point PCR reaction. For each genotype, one sample of synthesized first-strand cDNA was selected randomly and used for all primer tests. The master mix for this reaction was prepared using the 2 × Rapid Taq Master Mix (Vazyme Biotech Co., Ltd., Nanjing, China, Cat. No. R222) per the manufacturer’s instructions. The amplification was carried out using the following thermal cycling parameters: initial denaturation at 95 °C for 5 min, followed by 30 cycles of denaturation at 94 °C for 30 s, annealing for 30 s at the primer pair’s average annealing temperature, and an extension of 45 s at 72 °C. After completion of the last cycle, a final extension was carried out at 72 °C for 5 min^19^. To confirm that a single product was amplified, the PCR products were loaded into a 1.5% agarose-TAE gel stained with ethidium bromide and run at 120 V for 15 min at room temperature. Gel images were obtained using Gel Doc XR+ (Bio-Rad, USA).

### RT‑qPCR analyses

RT-qPCR assays were performed in a 20 μL reaction system using the ChamQ Universal SYBR qPCR Master Mix kit (Vazyme Biotech Co., Ltd., Nanjing, China, Cat. No. Q711–02/03) per the manufacturer’s protocol. Candidate genes were selected for RT-qPCR assays using an Applied Biosystems 7500 RT-qPCR System (Life Tech, 81 Wyman Street, Waltham, MA, 02454, USA). The thermal cycling conditions were as follows: 50 °C for 2 min; 95 °C for 10 min; 40 cycles at 95 °C for 15 s; 56 °C for 30 s; and 72 °C for 40 s. The melting curve was generated by heating the amplicon from 60 to 95 °C to confirm primer specificity. Three biological replicates were carried out for each PCR reaction. Relative fold changes in gene expression were calculated using the comparative 2^−ΔΔCT^ method^28^.

### Analysis of gene expression stability

NormFinder, geNorm, and BestKeeper were used to evaluate the expression stability of the reference genes in *N. dimidiatum* at various development stages, at different temperatures, and during interaction with pitaya.

The geNorm program determines the stability and optimal number of genes required to calculate the M-value and pairwise variation Vn/Vn + 1 between two sequential normalization factors^29^. The optimal candidate reference genes were determined based on the average expression stability between samples and the results of variance analysis. The BestKeeper program was used to identify stable reference genes, which calculates pairwise correlations based on values of standard deviation (SD) and percentage covariance (Cov)^30^.

### Validation of reference genes

To verify the reliability of the selected reference genes. Pectate lyase^31^, an enzyme involved in the digestion of the cell wall of pitaya, was analyzed using RT-qPCR in the early stages of *N. dimidiatum* infection of pitaya. The specific primers used are shown in Table 1.

## Supplementary Information

Supplementary Information 1.

## Data Availability

All data generated and analyzed during the study is included in the published article and its Supplementary Information files.
