# Supplementary material for: Identification of RT-qPCR reference genes suitable for gene function studies in the pitaya canker disease pathogen Neoscytalidium dimidiatum
Source: Sci Rep. 2022 Dec 26;12:22357. doi: 10.1038/s41598-022-27041-w (PMC9792573; doi:10.1038/s41598-022-27041-w)
Supplement: Supplementary file 1 — Supplementary Information 1. [file 41598_2022_27041_MOESM1_ESM.pdf]

# Identification of RT-qPCR reference genes suitable for gene function studies in the pitaya canker disease pathogen *Neoscytalidium dimidiatum*

Meng Wang[1,2], Zhouwen Wang[1,2], Shuangshuang Wei[2], Jun Xie[2], Jiaquan Huang[1,2], Dongdong Li[1,2], Wenbin Hu[3], Hongli Li[3], Hua Tang[1,2 *]

1. Sanya Nanfan Research Institute of Hainan University, Hainan Yazhou Bay Seed Laboratory, Sanya, China, 572025;

2. College of Tropical Crops/Hainan Key Laboratory for Sustainable Utilization of Tropical Bioresources, Hainan University, Haikou, China, 570228;

3. Tropical Crops Genetic Resources Institute, Chinese Academy of Tropical Agricultural Sciences, Haikou, China, 571700

*Corresponding author: Hua Tang. E-mail address: thtiger@163.com

Supplementary figures and tables

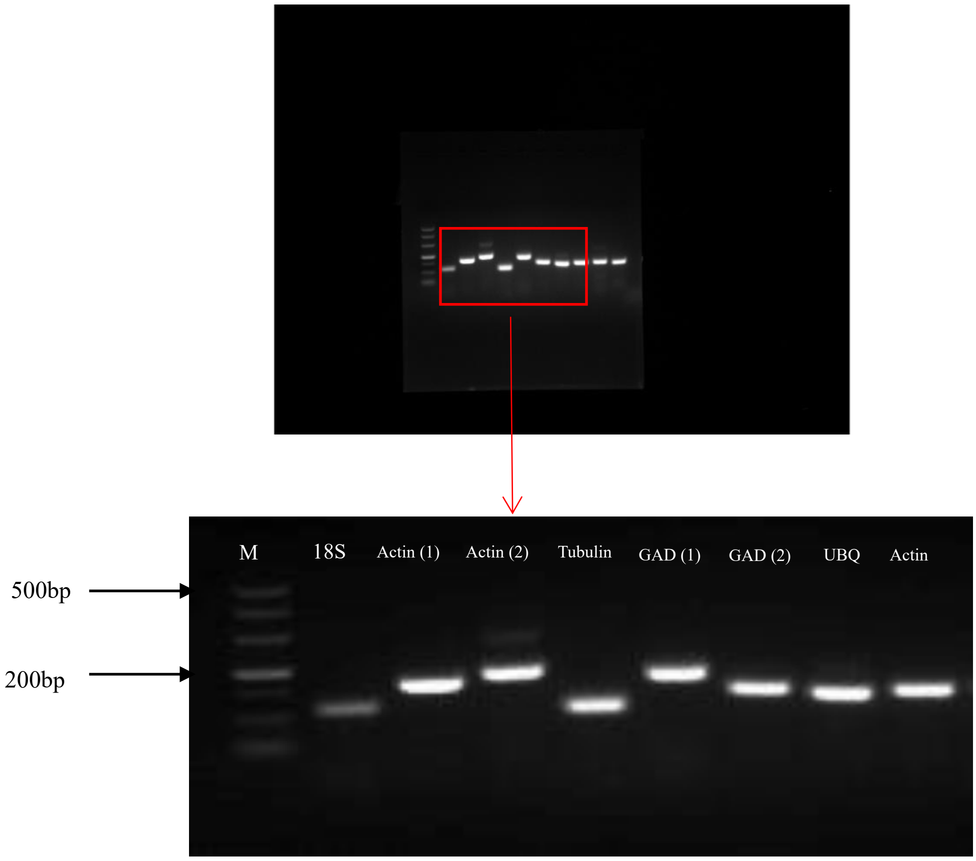


Figure S1. Agarose gel electrophoresis detection for specificity of RT-qPCR products. The results showed specific RT-qPCR products for each gene with expected size. M lane was DNA marker.

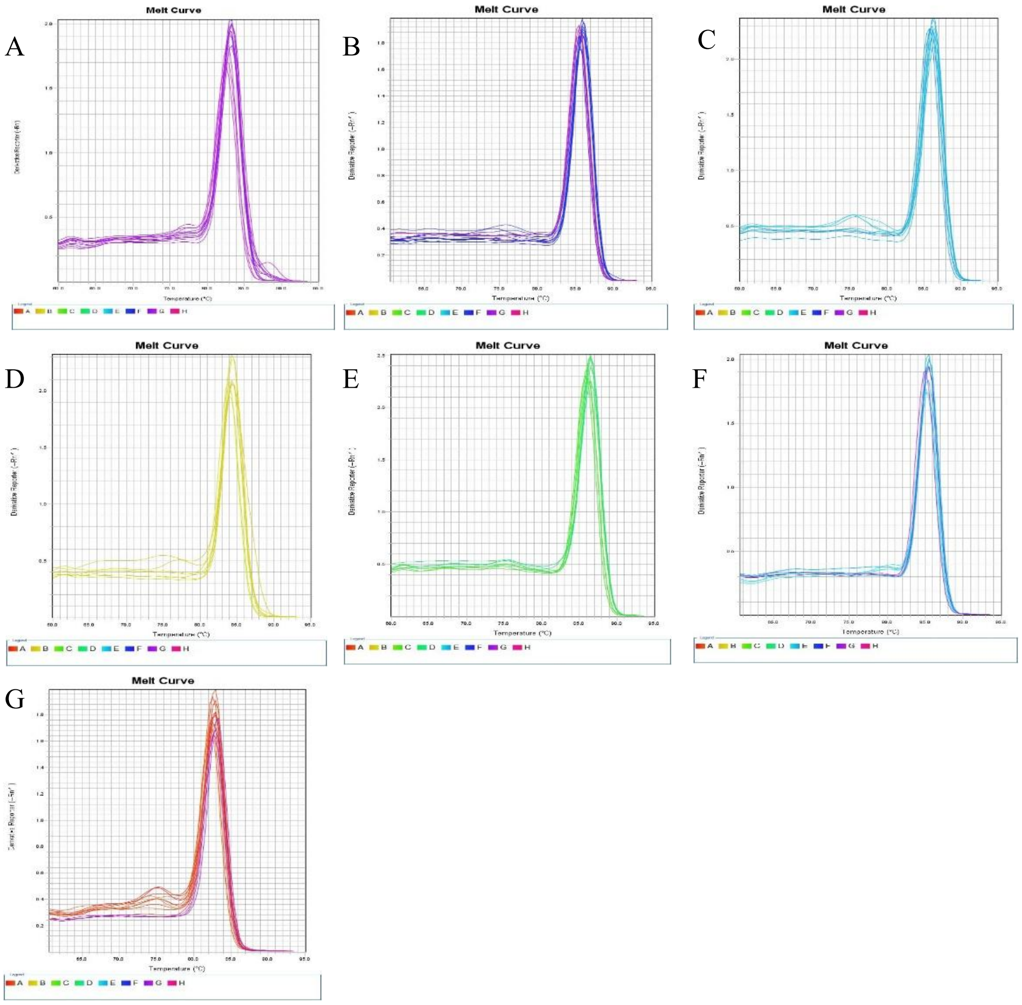


Figure S2. Melting curves of RT-qPCR for all the candidate reference genes. A: *UBQ*, B: *Actin*, C: *Actin (1)*, D: *Tubulin*, E: *GAPDH (1)*, F: *GAPDH (2)*, G: *18S rRNA*.

**Table S1.** Sequence information of *pectinase* genes from *Neoscytalidium dimidiatum*.

**Gene abbreviation-***pectinase*. **Gene ID-**ND3060. **Length-**759 bp.

**Gene sequence -**

ATGTTCTCCAAGCTTTTCCTGCTCCCCCTCCTGGCGGCTTCCGCCCTGGCTGCTCCTGC
CGACGACACTTTCGGCTACGAGCTCGTTCGCCGTGCGAACTTCCCTATCCCTGCCTCCA
AGGGAACTGTCAAGTACAGCTCTGCCAAGACCATCTCCGGCACCTTCGATGGTGGCTT
GAAGACCTACGGCCGTGGTGTCAAGTGCACTGGCCAGGCCGAGGGTGGTGACAAGGA
TGCCGTTTTCATCCTCGAGAACGGGGCCACCCTGAAGAACGCCATCATTGGCGCCGAC
CAGATCGAGGGTGTCCACTGCAAGGGCTCCTGCACCATCGAGAACGTCTGGTGGGCC
GCGGTCTGCGAGGACGCTCTCTCCCTGAAGGGCGACGGCAACGCCAAGGTTATTGGC
GGTGGTGCCACCGGCGCCGACGACAAGGTCATTCAGCACAACGGTATCGGCTCCGTTT
CCATCGACGGCTTCACCGTCGCCGACTTCGGCAAGCTCTACCGCTCTTGCGGTAATTGC
AAGGGTAACGGCGGCAAGCGTACTGTCACCATCAAGAACGTCAAGGCCTCCAACGGC
AAGCTCCTCGCTGGTATCAACTCCAACTACGGCGACACTGCTACCATCACCGGCACTT
GCGCTACCTCCGTCAAGAAGGTCTGCACTGAGTTCAAGGGCAACAACAACGGCAAGG
AGCCCACCGAGATCAGCTCCGGCTCCAGCAACGCCTGCAAGTACTCTTCCATCAAGGC
TTGCTAG
